# Supplementary material for: Personalizing chemotherapy drug selection using a novel transcriptomic chemogram
Source: PLoS Comput Biol. 2025 Sep 10;21(9):e1013417. doi: 10.1371/journal.pcbi.1013417 (PMC12449002; doi:10.1371/journal.pcbi.1013417)
Supplement: S2 File — (PDF) [file pcbi.1013417.s002.pdf]

Gene	Frequency	Cis	Cyta	5-FU	Gem	Irino	Lumi	Pac	Topo	Vinb	Vor
MMP10	6	✓	✓			✓		✓	✓	✓	
ADAT2	5	✓	✓	✓				✓		✓	
NPM3	5	✓	✓	✓				✓		✓	
C1QBP	4	✓		✓				✓		✓	
RIOK1	4	✓						✓		✓	✓
SLFN11	4	✓			✓	✓			✓		
KRT5	3	✓						✓	✓		
LY6K	3	✓				✓			✓		
CLYBL	3		✓	✓							✓
SLC27A5	3		✓	✓				✓			
TAF4B	3		✓					✓		✓	
COQ3	3			✓				✓		✓	
CSTA	3			✓				✓		✓	
FRAT2	3			✓				✓			✓
NRARP	3			✓				✓			✓
SOX7	3					✓		✓	✓		
WDFY2	3						✓	✓	✓		
C15orf41	2	✓								✓	
CDCA7	2	✓						✓			
LRRC8C	2	✓					✓				
PSAT1	2	✓	✓								
STOML2	2	✓								✓	
USP31	2	✓						✓			
ZNF750	2	✓						✓			
ACN9	2		✓							✓	
ASNS	2		✓							✓	
CCNB1IP1	2		✓							✓	
F12	2		✓	✓							
FASTKD1	2		✓	✓							
MYC	2		✓					✓			
POLR1D	2		✓	✓							
SFXN4	2		✓	✓							
DSG3	2			✓				✓			
MYB	2			✓							✓
UQCRH	2			✓						✓	
POLR3G	2				✓		✓				
AIM2	2					✓			✓		
BNC1	2					✓	✓				
FOXL2	2					✓			✓		
ITPRIP	2					✓	✓				
SERPINB4	2					✓			✓		
JARID2	2						✓	✓			
TAF5	2							✓		✓	
TMEM206	2							✓		✓	
ATP1B3	1	✓									
CDC7	1	✓									
FKBP14	1	✓									
WDR3	1	✓									
C12orf57	1		✓								
DLEU1	1		✓								
DPH5	1		✓								

Gene	Frequency	Cis	Cyta	5-FU	Gem	Irino	Lumi	Pac	Topo	Vinb	Vor
FAR1	1		✓								
GNPNAT1	1		✓								
MTHFD2	1		✓								
NOB1	1		✓								
SIGMAR1	1		✓								
SNRPA1	1		✓								
TUBE1	1		✓								
ATP5D	1			✓							
CHCHD10	1			✓							
FAM83F	1			✓							
GMDS	1			✓							
MRPL2	1			✓							
MUC13	1			✓							
PDSS1	1			✓							
PIP5K1B	1			✓							
PPP1R1B	1			✓							
REG4	1			✓							
RPL22L1	1			✓							
SPINK4	1			✓							
VSNL1	1			✓							
ZNF511	1			✓							
ARNTL2	1				✓						
CRLF3	1				✓						
CXCL1	1				✓						
ELK3	1				✓						
GLIPR1	1				✓						
MLKL	1				✓						
PROCR	1				✓						
RELB	1				✓						
BCL2A1	1					✓					
HMGA1	1					✓					
PGM2	1					✓					
SLC6A15	1					✓					
TRAF3	1					✓					
ADAMTS6	1						✓				
ARHGAP22	1						✓				
CDH13	1						✓				
CSGALNACT2	1						✓				
CTHRC1	1						✓				
DSE	1						✓				
DYNC2H1	1						✓				
DZIP1	1						✓				
FAM101B	1						✓				
IL27RA	1						✓				
KRT14	1						✓				
MFAP2	1						✓				
PDLIM4	1						✓				
POPDC3	1						✓				
RFTN1	1						✓				
SH3PXD2B	1						✓				
SLC31A2	1						✓				

Gene	Frequency	Cis	Cyta	5-FU	Gem	Irino	Lumi	Pac	Topo	Vinb	Vor
SLC4A7	1						✓				
SPHK1	1						✓				
TM4SF19	1						✓				
TWIST1	1						✓				
VEGFC	1						✓				
IL1B	1								✓		
RAB38	1								✓		
C6orf170	1									✓	
ECSIT	1									✓	
FGF11	1									✓	
FSD1	1									✓	
LRRC49	1									✓	
NOC3L	1									✓	
RPF2	1									✓	
SEH1L	1									✓	
TBPL1	1									✓	
ARID3B	1										✓
C9orf152	1										✓
CECR5	1										✓
DCXR	1										✓
DQX1	1										✓
EFNA3	1										✓
FHIT	1										✓
FKBP4	1										✓
IL17RB	1										✓
JHDM1D	1										✓
NOC2L	1										✓
PDCD2L	1										✓
PEX7	1										✓
PHGR1	1										✓
SDHAF1	1										✓
TIMM8B	1										✓
TMEM168	1										✓
TMEM183A	1										✓
TOX3	1										✓
TRAP1	1										✓
TTC39A	1										✓
AMTN	1							✓			
ARTN	1							✓			
C1orf74	1							✓			
CYB5R4	1							✓			
FAT2	1							✓			
GBP6	1							✓			
GNA15	1							✓			
GPC2	1							✓			
HOXD10	1							✓			
KRT6A	1							✓			
KRT6B	1							✓			
MARK1	1							✓			
MMP13	1							✓			
PKP1	1							✓			

Gene	Frequency	Cis	Cyta	5-FU	Gem	Irino	Lumi	Pac	Topo	Vinb	Vor
PREP	1							✓			
PVRL1	1							✓			
REL	1							✓			
S100A7	1							✓			
SH3BP1	1							✓			
TP63	1							✓			
TRERF1	1							✓			
