# Supplementary figures and images for: Personalizing chemotherapy drug selection using a novel transcriptomic chemogram
Source: PLoS Comput Biol. 2025 Sep 10;21(9):e1013417. doi: 10.1371/journal.pcbi.1013417 (PMC12449002; doi:10.1371/journal.pcbi.1013417)

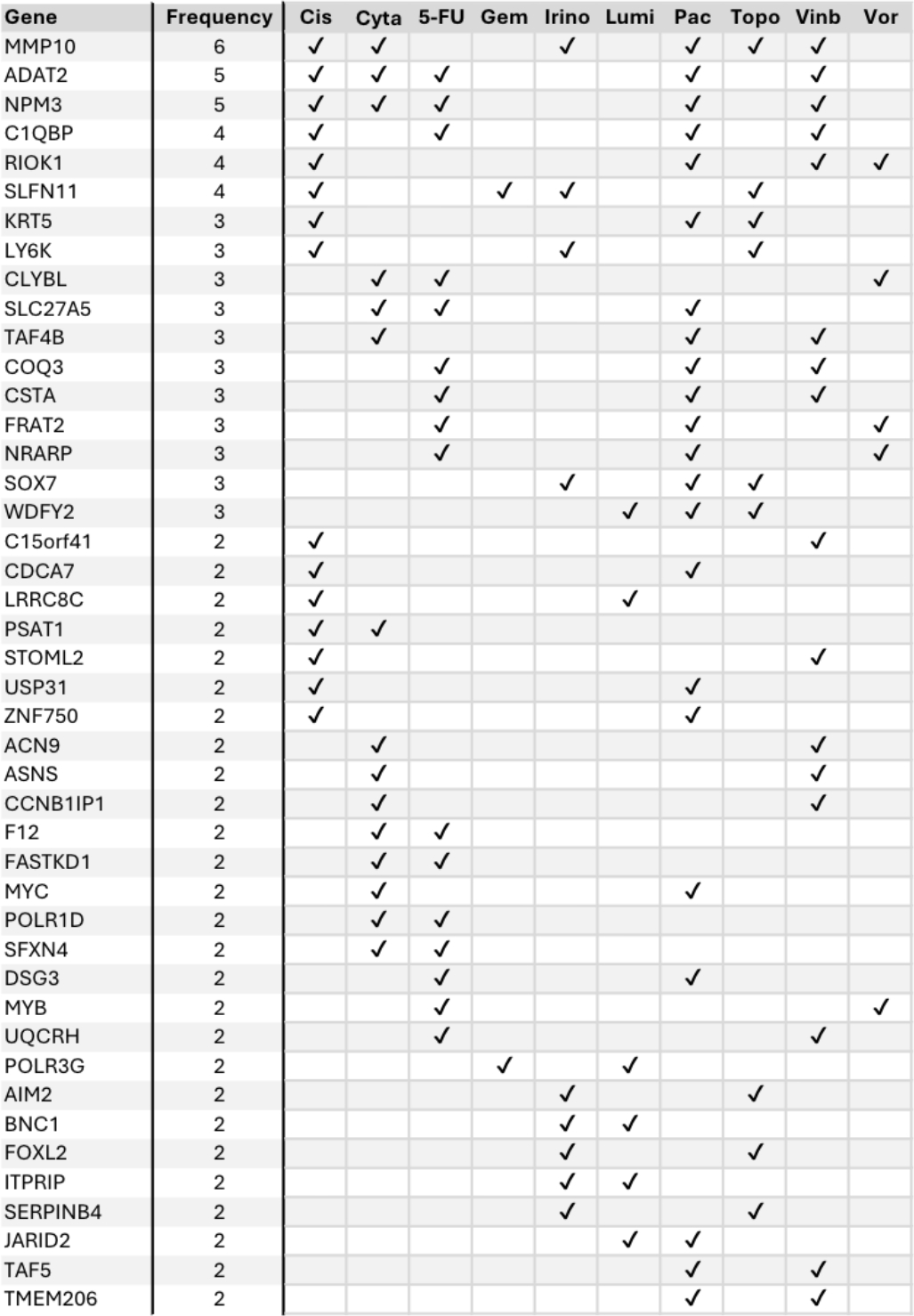

Supplement: S1 Fig — Genes that appear in more than one of the 10 signatures used are listed. Out of 160 unique genes across 10 signatures, 44 are present in multiple signatures. For the full table including genes that appear in only 1 signature, see Supplemental File 2. To see entire signatures listed out per drug, see Supplemental File 1. (TIFF) [file pcbi.1013417.s003.tif]

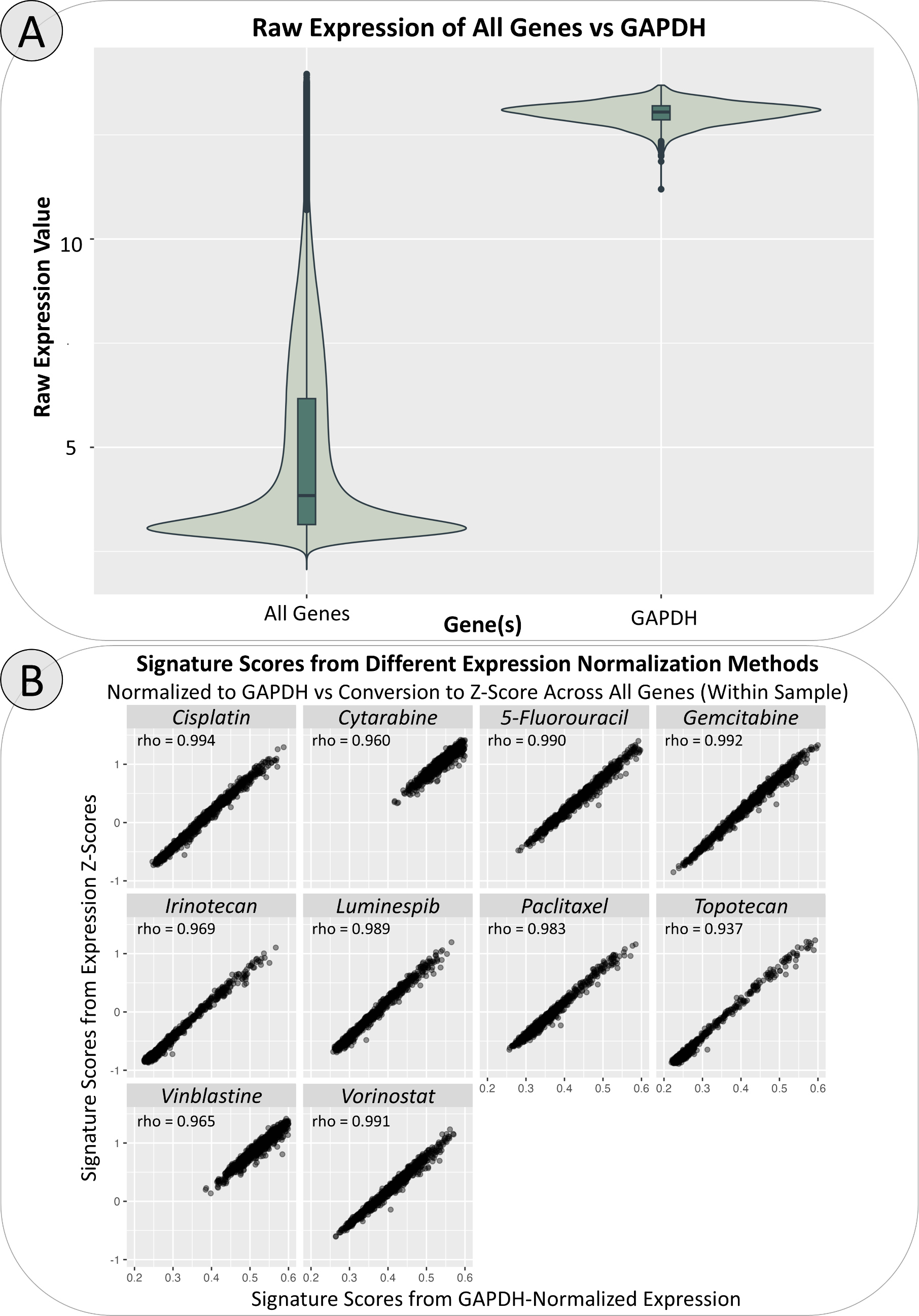

Supplement: S2 Fig — A. Among all cell lines in GDSC, GAPDH consistently exhibits high expression levels. As such, gene expression (for use within the chemogram) can be normalized to GAPDH expression within each cell line rather than normalizing by converting expression values to z-scores. B. Signature scores derived for all drugs and cell lines are compared when calculated using GAPDH-normalized expression or expression z-scores. Signature scores derived from either normalization method are highly correlated for all signatures. Spearman’s rho is indicated at the top of each subpanel. (TIFF) [file pcbi.1013417.s004.tif]

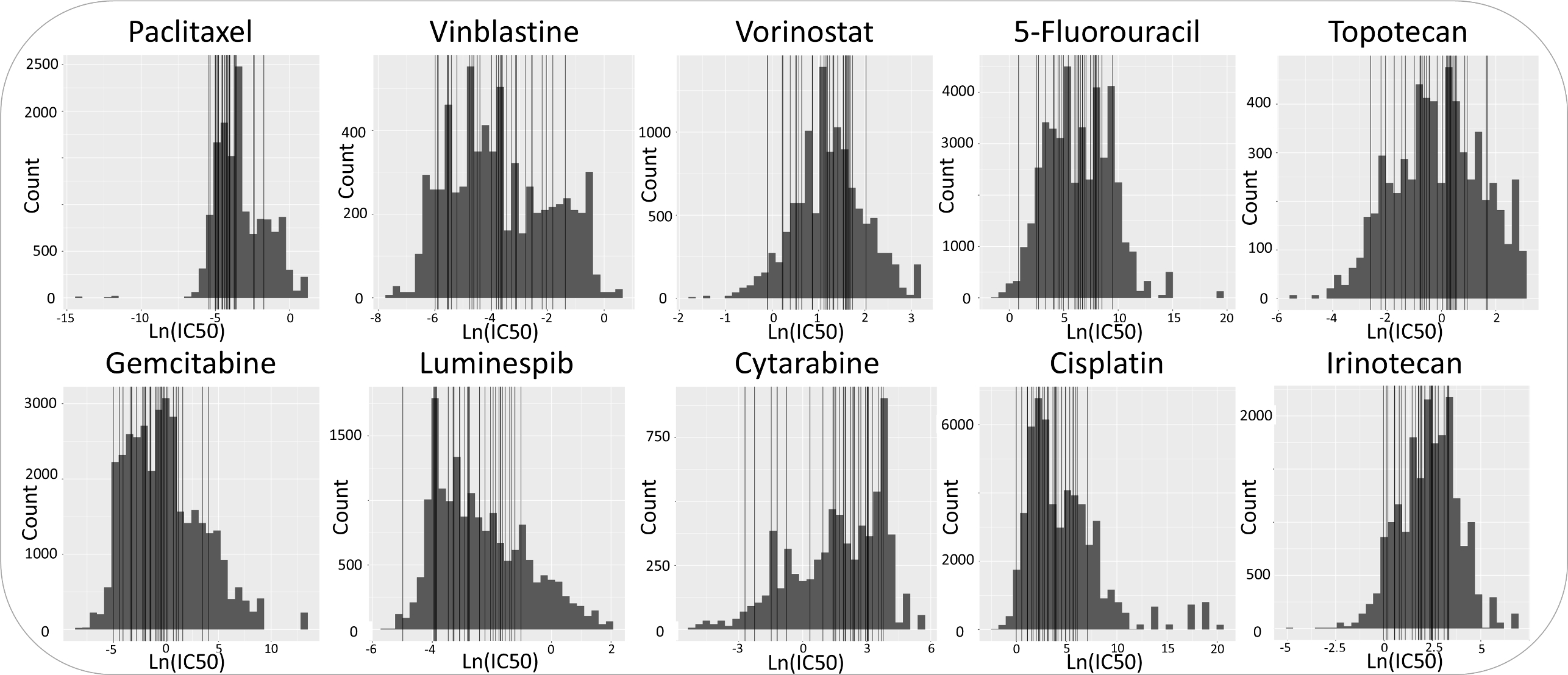

Supplement: S3 Fig — The natural log of IC50s calculated using the gdscIC50 package are shown in the histograms. Vertical black lines denote the standard doses used for validation as described in Fig 3. (TIFF) [file pcbi.1013417.s005.tif]

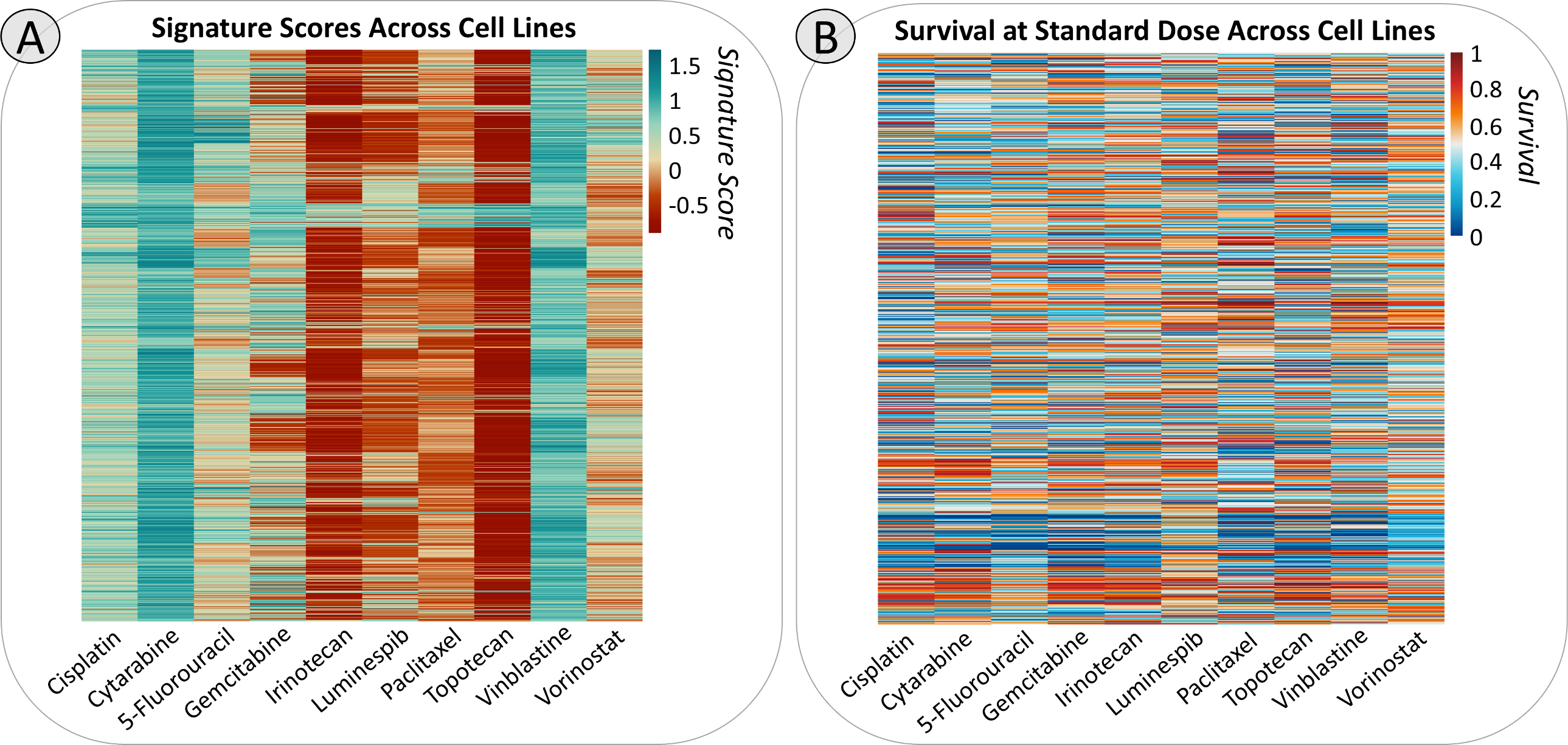

Supplement: S4 Fig — A. Signature scores for all cell lines are calculated for all 10 gene signatures. Each column depicts the signature scores generated from one signature, and each row represents a single cell line. The higher the score, the greater the predicted sensitivity. Signature scores across cell lines tend to be similar within drugs. B. The fraction of surviving cells was measured at standardized doses against each of the 10 drugs. Each column represents survival against one drug, and each row represents a single cell line in the same order as shown in panel B. A higher survival indicates greater resistance against a drug, and a lower survival indicates greater sensitivity against a drug. (TIFF) [file pcbi.1013417.s006.tif]

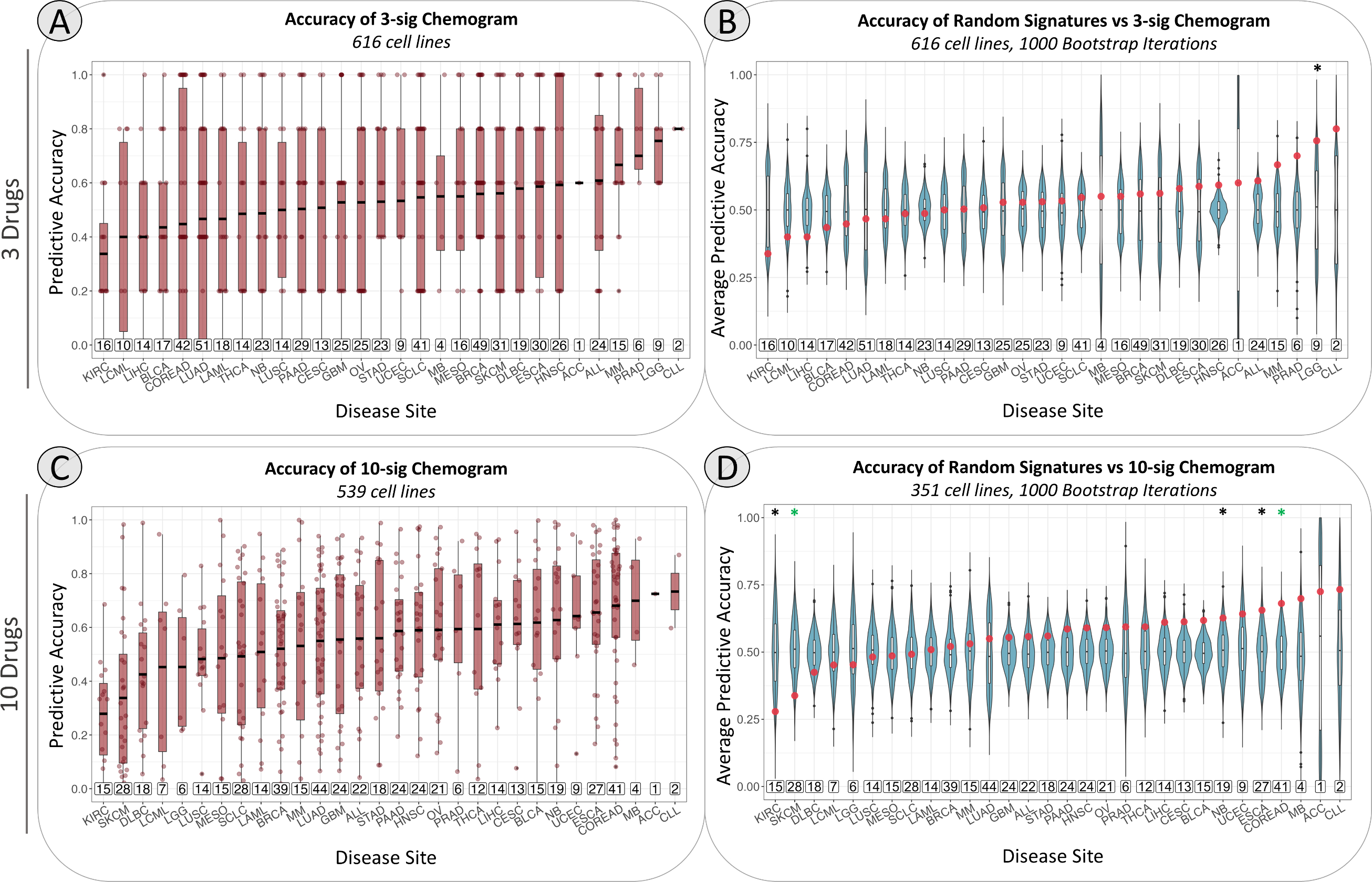

Supplement: S5 Fig — This figure is formatted the same as Fig 6, but includes both epithelial and non-epithelial cancer cell lines. Cancer subtypes are abbreviated using TCGA study abbreviations. The boxed numbers above the x-axis indicate the number of cell lines included in each cancer type. The lines in the center of the boxplots represent the mean accuracy, rather than the median. Asterisks indicate p ≤ 0.05 via unpaired Wilcoxon rank sum test. Green asterisks indicate FDR-adjusted p ≤ 0.05. The results are very similar to that of only the epithelial cancers for both the 3-drug and 10-drug chemograms. (TIFF) [file pcbi.1013417.s007.tif]

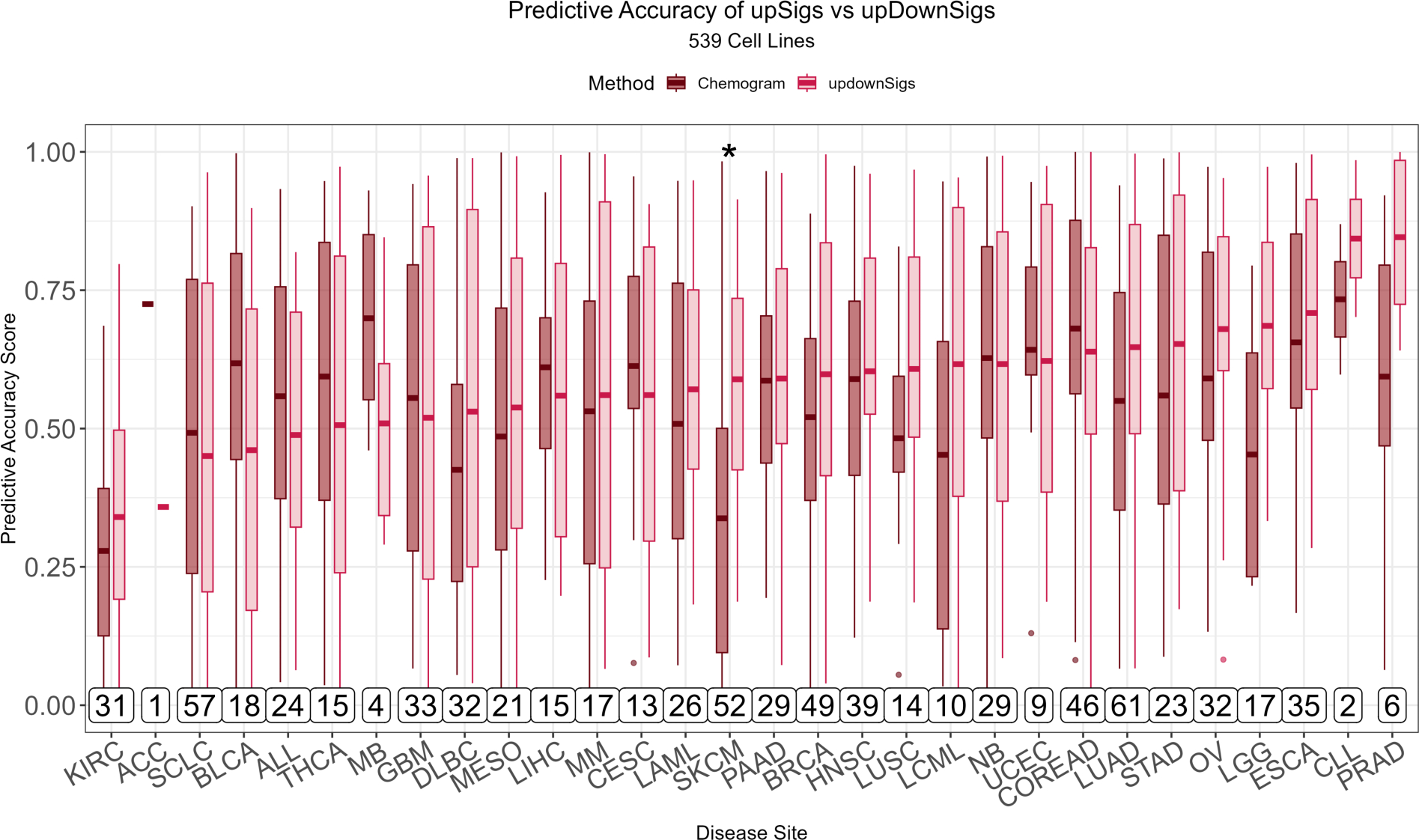

Supplement: S6 Fig — Cancer subtypes are abbreviated using TCGA study abbreviations. Both epithelial-origin and non-epithelial-origin cancers are included. The boxed numbers above the x-axis indicate the number of cell lines included in each cancer type. The lines in the center of the boxplots represent the mean accuracy, rather than the median. Each point represents the predictive accuracy scores for a cell line. The red boxplots correspond to the accuracy scores of the chemogram using our up-regulated signatures only, while the pink boxplots correspond to the accuracy scores of the chemogram using both up- and down-regulated signatures. With α = 0.05, only skin cutaneous melanoma (SKCM) had significant differences between method performance based on a two-tailed Wilcoxon rank-sum test with either a Bonferroni-corrected threshold or with an FDR-corrected p-value. (TIFF) [file pcbi.1013417.s008.tif]

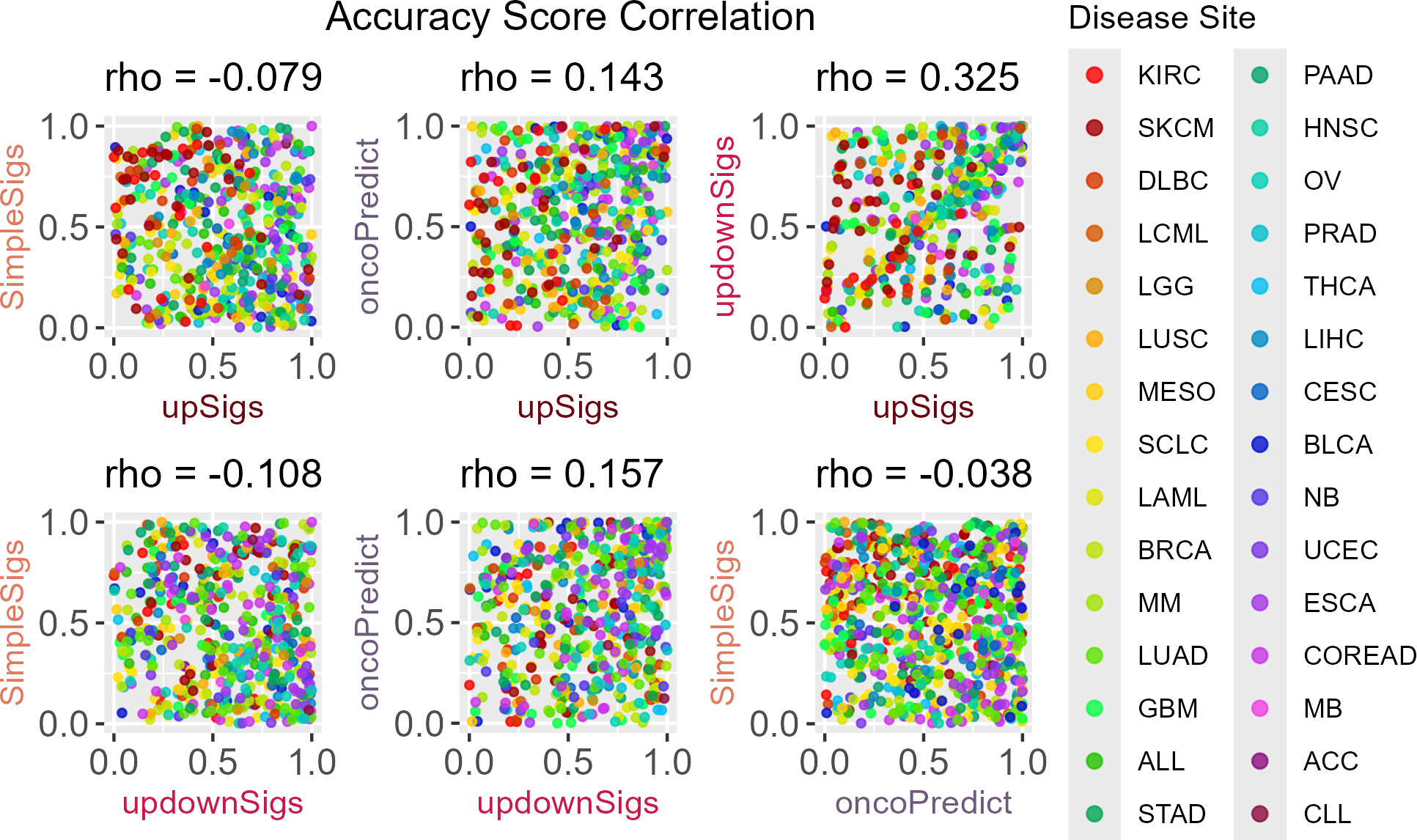

Supplement: S7 Fig — The same plot as Fig 7B is shown here, but with each point colored by the cancer subtype associated with the cell line being presented. No major trends are apparent among any particular cancer subtype, although accuracy scores for SKCM from the up- and down-regulated signatures together are noticeably higher than the up-regulated signatures alone. (TIFF) [file pcbi.1013417.s009.tif]

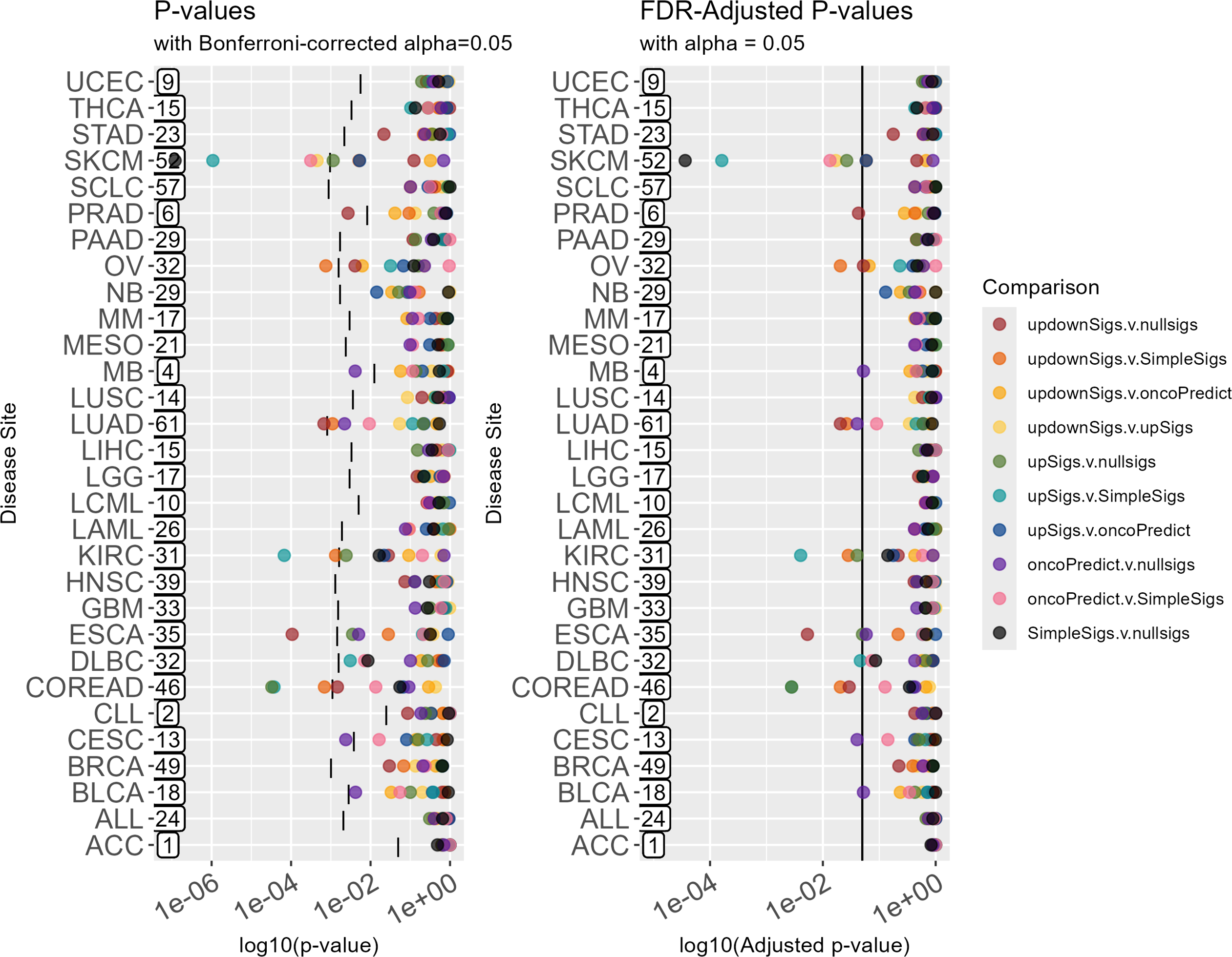

Supplement: S8 Fig — Pairwise comparisons among all methods per cell line were made using two-tailed Wilcoxon rank-sum tests. P-values for each comparison are shown as individual points, with color corresponding to the comparison being made. Log10(p-value) is shown on the x-axis of the left panel, while log10(false discovery rate-adjusted p-value) is shown on the x-axis of the right panel. The y-axis denotes cancer type, and the boxed number to the right of the y-axis shows the number of cell lines included in the associated cancer type. In the left panel, Bonferroni-corrected significance thresholds are marked with a vertical black line at α = 0.05 divided by the number of cell lines in the cohort. In the right panel, a black line marks the significance threshold of α = 0.05. (TIFF) [file pcbi.1013417.s010.tif]
