# Supplementary material for: Personalizing chemotherapy drug selection using a novel transcriptomic chemogram
Source: PLoS Comput Biol. 2025 Sep 10;21(9):e1013417. doi: 10.1371/journal.pcbi.1013417 (PMC12449002; doi:10.1371/journal.pcbi.1013417)
Supplement: Attachment — Submitted filename: review.pdf [file pcbi.1013417.s011.pdf]

Summary

The authors present chemogram, a method to derive signatures from cell lines' gene expression data which are indicative of the response to chemotherapeutic agents. The method is based on differential gene expression analysis between groups of sensitive and non-sensitive cell lines for a given agent. The calculated signatures are used to rank drugs from most to least effective against a given cell line. The predicted rankings are then compared with observed ones. Within most cancer types, the predictions yielded by chemogram are better than ones stemming from randomly generated signatures. The results are presented for two sets of drugs, one comprising 3 and other comprising 10 drugs.

The paper is relatively well-written and clearly structured. Visualizations are clear. Code to recreate the analysis and compute the signatures is provided. The provided table with derived signature genes for 26 drugs is a potentially valuable addition.

Major comments

The main issue is that the results showing the potential utility of the proposed method are unconvincing. The only comparison is to the randomly generated signatures. According to the authors, in the case of 3-drugs set, chemogram beats null distribution only in neck squamous cell carcinoma and prostate adenocarcinoma (are these results statistically significant? Because there are no asterixes there in Fig. 6B). For many cancer sites, the chemoram exhibits the same or worse performance than null distribution. For the 10-drugs set, statistically better results are also only produced in esophageal carcinoma and colorectal adenocarcinoma, while being worse for kidney renal clear cell carcinoma (KIRC). What are the global results, when pooling cell lines from all cancer types together?

The paper lacks sufficient comparison with other approaches besides random signatures in order to make valid claims about the utility of chemogram. The method could be compared with other methodologies to derive gene expression signatures or with simpler, but not random approaches (e.g. signature based on top genes with highest absolute correlation between expression and response). Also, the authors claim they cannot compare to existing machine learning models since those predict response for single drug rather than yielding ranking of the drugs. However, these predictions can be easily converted to ranking by generating predictions for a given cell line for all drugs of interest, and ranking the drugs according to the corresponding predicted value. Other published, sophisticated models could be used for comparison, but also simple ML models built by authors, even with the same features (genes) derived from the signatures, or some other baseline features set would enrich the comparison. However, no such baseline was produced, making the results not convincing.

From the description of the method, I assume the final score is calculated by taking the median expression of chosen upregulated genes. What about downregulated genes? If the authors claim that there is no benefit of considering downregulated genes, it is not justified in the paper. Producing a single score from both upregulated and downregulated genes would be harder, but it might improve the method.

The novelty of the method is questionable, since it is essentially a composition of three differential gene expression analyses and co-expression analysis.

Minor comments

The choice of showing results for a set of 3 drugs and a set of 10 drugs is not entirely clear to me. Was 3 drugs supposed to reflect the more real-life situation in which health providers need to choose among 3 candidates which are usually used to treat a given cancer type? But then shouldn't we have different sets for different types of cancer? How were the 3 drugs chosen? The 10 drugs were picked out of 26 because corresponding signatures exhibited best results, but on what criteria were those 26 chosen among hundreds from GDSC?

The formula for calculating accuracy between two rankings in Fig. 5 is not explained. In this form, it is unclear, and we also don't know how it changes with more than three drugs. It is also quite unintuitive. Maybe other methods to compare rankings would be more suitable - if not, why this one?
