# Supplementary material for: Personalizing chemotherapy drug selection using a novel transcriptomic chemogram
Source: PLoS Comput Biol. 2025 Sep 10;21(9):e1013417. doi: 10.1371/journal.pcbi.1013417 (PMC12449002; doi:10.1371/journal.pcbi.1013417)
Supplement: Attachment — Submitted filename: Response to Reviewers.pdf [file pcbi.1013417.s012.pdf]

Cleveland Clinic
Department of Translational Hematology and Oncology Research
9500 Euclid Avenue, Cleveland Ohio, 44195, US
June 23, 2025
e-mail: scottj10@ccf.org

Dear Dr. Robinson-Rechavi & Dr. Ioshikhes,,

We thank you and the anonymous reviewers for the review of our work and for the opportunity to improve it. To this end, we would like to resubmit our manuscript (PCOMPBIOL-D-25-00615) titled "Personalizing chemotherapy drug selection using a novel transcriptomic chemogram". Please find appended a point-by-point response to the thoughtful and constructive comments from the reviewers. Please note that all changes will appear in blue text in the revised document.

Sincerely,

Jacob Scott, on behalf of our coauthors

Reviewer 1 writes:

The authors present chemogram, a method to derive signatures from cell lines' gene expression data which are indicative of the response to chemotherapeutic agents. The method is based on differential gene expression analysis between groups of sensitive and non-sensitive cell lines for a given agent. The calculated signatures are used to rank drugs from most to least effective against a given cell line. The predicted rankings are then compared with observed ones. Within most cancer types, the predictions yielded by chemogram are better than ones stemming from randomly generated signatures. The results are presented for two sets of drugs, one comprising 3 and other comprising 10 drugs. The paper is relatively well-written and clearly structured. Visualizations are clear. Code to recreate the analysis and compute the signatures is provided. The provided table with derived signature genes for 26 drugs is a potentially valuable addition.

Major Comments:

1. *The main issue is that the results showing the potential utility of the proposed method are unconvincing. The only comparison is to the randomly generated signatures... The paper lacks sufficient comparison with other approaches besides random signatures in order to make valid claims about the utility of chemogram. The method could be compared with other methodologies to derive gene expression signatures or with simpler, but not random approaches (e.g. signature based on top genes with highest absolute correlation between expression and response). Also, the authors claim they cannot compare to existing machine learning models since those predict response for single drug rather than yielding ranking of the drugs. However, these predictions can be easily converted to ranking by generating predictions for a given cell line for all drugs of interest, and ranking the drugs according to the corresponding predicted value. Other published, sophisticated models could be used for comparison, but also simple ML models built by authors, even with the same features (genes) derived from the signatures, or some other baseline features set would enrich the comparison.*

   We thank the reviewer for emphasizing this shortcoming of our study and for suggesting alternative methods to compare our model to. To accommodate for this, we've tested a variety of additional predictive models in a chemogram format to rank predicted sensitivity, and these results are shown in a new section titled "Comparison of other predictive models in a chemogram format" along with Figure 7 and Supplemental Figures 6-8. For these method comparisons, we use these alternative models to predict drug response among the same 10 drugs as before, then ranked those predictions per individual sample. The remaining analyses remain the same as the prior analyses (comparing to observed survival at a standardized dose per cancer type, then comparing these observed sensitivity rankings to the predicted sensitivity ranking and scoring for accuracy as per Figure 5).

   We first tested a simpler predictive signature model, extracting these signatures using a single differential expression analysis method and no cross-validation or patient sample co-expression filtering. These signatures performed only slightly better than the null distribution, with a global average accuracy of only 0.53.

   Next, we used a method published by Maeser *et al* called *oncoPredict*. This model uses Ridge Regression trained on GDSC cell line drug response and gene expression as well as clinical gene expression data gathered from various publications. The output of this model is imputed IC_{50} values, and no relative drug

sensitivity rankings are produced. In order to generate rankings and compare this predictive model to ours, we converted the imputed IC_{50}s to z-scores across all GDSC cell lines for each drug, then used the z-scores to rank relative drug sensitivity within cell lines. The global accuracy scores were very similar to that of our original method (global average accuracy of 0.54 for oncoPredict and 0.55 for our method). Although the global performance of oncoPredict is very similar to our method, the predictions are not correlated per cell line or per cancer type, making it difficult to parse out specific cases where this model would be superior over ours and vice versa.

We also compared the performance of our original method (using gene signatures whose up-regulation is associated with drug sensitivity) to an alternative version that integrates both up- and down-regulated gene signatures. The results of this will be described later in this rebuttal.

2. *According to the authors, in the case of 3-drugs set, chemogram beats null distribution only in neck squamous cell carcinoma and prostate adenocarcinoma (are these results statistically significant? Because there are no asterixes there in Fig. 6B).*

   To confirm the reviewer's observation, no – the difference between the null distributions for the 3-signature chemogram in neck squamous cell carcinoma and prostate adenocarcinoma are not statistically significant. As such, the lack of asterisks here are intentional.

3. *What are the global results, when pooling cell lines from all cancer types together?*

   We thank the reviewer for this inquiry and agree that showing global results is constructive, especially for comparing different models (real signatures vs. null signatures) used in the chemogram format. We show these results in Figure 7a, along with pooled results for other predictive methods. Using a two-tailed Wilcoxon rank-sum test, there are no statistically significant differences between pooled samples, but there are numerous subtype-specific cases that show significant differences between methods as shown in Supplemental Figure 8.

4. *From the description of the method, I assume the final score is calculated by taking the median expression of chosen upregulated genes. What about downregulated genes? If the authors claim that there is no benefit of considering downregulated genes, it is not justified in the paper. Producing a single score from both upregulated and downregulated genes would be harder, but it might improve the method.*

   Correct, drug sensitivity for an individual drug is summarized by taking the median expression of the signature genes. Originally, we focused on demonstrating our method only using genes that were up-regulated in sensitive samples because gene up-regulation, as opposed to down-regulation, is more reliably detectable and tends to be more statistically robust. In light of this excellent comment, we decided to pursue the reviewer's suggestion and summarize drug sensitivity by calculating a single score from both up- and down-regulated gene signatures. Indeed, this approach noticeably improved the method.

   To extract the down-regulated signatures, we used the same Scarborough *et al* pipeline as before with the same parameters, but this time filtered for only

the down-regulated genes in sensitive samples. To integrate these additional biomarkers with the original chemogram methodology, drug sensitivity for individual drugs was summarized by subtracting the median expression of the down-regulated signature from the median expression of the up-regulated signature (up minus down). Prediction ranking and accuracy scoring were done in the same way as all previous analyses.

The addition of the down-regulated signatures to the up-regulated signatures yielded a noticeable improvement to global accuracy scores, with an average of 0.58 compared to 0.55 for the up-regulated gene signatures only, though this global difference was not statistically significant. Accuracy scores stratified by cancer subtype can be seen in Supplemental Figure 6. From this figure, as well as Figure 7b and Supplemental Figure S7, it is evident that in certain disease sites (such as skin cutaneous melanoma), the addition of down-regulated gene signatures can yield substantial improvements to ranking predicted drug sensitivity. Conversely, there are a few cases (such as bladder cancer and cervical squamous cell carcinoma) where using only up-regulated gene signatures to rank predicted sensitivity would be superior to using both up- and down-regulated signatures, although these differences are not statistically significant.

Minor Comments:

1. *The choice of showing results for a set of 3 drugs and a set of 10 drugs is not entirely clear to me. Was 3 drugs supposed to reflect the more real-life situation in which health providers need to choose among 3 candidates which are usually used to treat a given cancer type? But then shouldn't we have different sets for different types of cancer? How were the 3 drugs chosen? The 10 drugs were picked out of 26 because corresponding signatures exhibited best results, but on what criteria were those 26 chosen among hundreds from GDSC?*

   We thank the reviewer for revealing that the explanation of this part of the methodology lacked clarity and have added commentary to the manuscript accordingly. The selection of 3 drugs to initially outline our method was arbitrary – it is a small enough number to clearly describe the step-by-step process of our study without making said descriptions overly lengthy. The selection of 10 drugs is elaborated on in the Methods section under "Sensitivity Signature Extraction". We sought to extract predictive signatures for many commonly used cancer drugs. Many of the drugs in GDSC were not able to produce gene signatures using our pipeline. As such, we filtered our signature list to only include those that predicted response to chemotherapies, leaving us with 26 signatures. For these remaining 26 signatures, we assessed the accuracy of each using the *in vitro* hazard ratio metric described by Scarborough *et al.* The performance of each signature was assessed by calculating the signature score (median z-score expression of the signature genes) for all GDSC cell lines, then comparing the distribution of IC_{50} between cell lines in the highest and lowest quintiles of the signature scores. From this, we then calculated a hazard ratio, and this metric was used to rank all 26 signatures from best to worst accuracy. The top 10 performing signatures were used in this study.

2. *The formula for calculating accuracy between two rankings in Fig. 5 is not explained. In this form, it is unclear, and we also don't know how it changes with more than three drugs. It is*

also quite unintuitive. Maybe other methods to compare rankings would be more suitable - if not, why this one?

We appreciate the reviewer's emphasis on the need for further explanation and justification for the accuracy scoring method. The scalability of the scoring method is indicated on the bottom left of Figure 5 – the number of possible scores increases with the number of possible permutations. To address the reviewer's comment, we further elaborated on the scoring method and why we prefer it over other relevant statistical methods, namely Kendall's tau, in the section "Using multiple signatures allows for the prediction of sensitivity rank order".

Kendall's tau is also a method of comparing ranked lists and is an established statistical method, but is more limited in the number of possible scores, making the resulting metric less descriptive. Using Kendall's tau to compare ranked lists of 3 drugs can only yield 4 possible values (number of possible outcomes = ((n(n-1))/2)+1), whereas our method can produce 6 possible scores (number of possible outcomes = n!). When comparing ranked lists of 10 drugs, as we do later in the study, Kendall's tau can take only 46 distinct values, while our approach can yield 3,628,800. Our method is also more clinically relevant because we assign specific scores to every possible permutation of predicted order, allowing us to assign a higher score when drugs predicted to be more effective are actually observed to be. This could have important clinical implications if a clinician were to give these drugs sequentially based on the predicted ranking. For instance, following the example shown in Figure 5, if the predicted sensitivity ranking is drug A, B, then C, it would be more beneficial for the patient if the actual sensitivity ranking was drug B, A, then C, as opposed to B, C, then A. Other established statistical methods for comparing rank order would not be able to quantify these subtle distinctions.

Reviewer 2 writes:

Lin-Rahardja and colleagues build upon an important recent study which described an algorithm for predicting drug sensitivity of human cancer cell lines using publicly available gene expression and drug sensitivity data from GDSC and CTRP (Scarborough et al, 2023, NPJ Precision Oncology). That prior work addressed an important unmet need to identify which patients are most likely to benefit from a particular chemotherapy, with the first proof of concept focused on cisplatin. This new article applies the previously described algorithm to 9 other cancer chemotherapies, nearly all of which are widely used in clinical oncology, and all of which lack clear biomarkers of response. Testing their method's applicability to this substantially expanded set of clinically significant chemotherapies is an important goal, especially given the lack of existing predictive biomarkers for these therapies. The introduction persuasively describes the need for greater precision in the choice of chemotherapies, and how a gene expression-based algorithm, if effective, has greater potential for widespread use than in vitro drug screening on biopsied material. The study develops tissue-agnostic 'chemograms' based on publicly available data. 5-fold cross-validation is a suitably rigorous means to classify resistant versus sensitive cell lines. Given the lack of consensus methods for calling differentially expressed genes, the authors apply multiple algorithms and select genes found in the intersection of all methods, which I anticipate should add to the robustness of results.

Predictive performance is assessed on a tissue-specific basis, which shows that 3-gene and 10-gene chemograms outperform random signatures for some tissue types and for some drugs. This is to be expected, and it would be unreasonable to expect the approach to be effective for all tissue types and all drugs (I would be skeptical if a study made such a claim).

1. *Defining the specific contexts where chemograms could be effective is the most important result of the study. As written, the results and conclusion sections emphasize the overall evidence for the predictive potential of chemograms. I think one or both sections would benefit from more specific statements about which drug classes and tissue types appear amenable to this approach, recognizing that it would be unreasonable to expect universal applicability, and indeed the evidence does not suggest universal applicability.*

   We thank the reviewer for this excellent suggestion. We discussed specific disease sites where our method could be most beneficial at the end of the section "Signature-based chemograms are scalable for any number of drugs", as well as throughout the new section "Comparison of other predictive models in a chemogram format". The most promising use-cases for the chemogram include esophageal carcinoma and colorectal adenocarcinoma. With the addition of down-regulated gene signatures, skin cutaneous melanoma could also be a context where the chemogram may be very useful.

2. *Distributions of IC50s in predicted sensitive and resistant cultures are compared by Hazard Ratio. As this is a non-standard use of the Proportional Hazards model, it would be helpful for the methods or discussion to concisely mention that these hazard ratios quantify in vitro differences and do not indicate or predict time-to-event hazard ratios in humans.*

   We thank the reviewer for their suggestion to improve clarity. The calculation of the hazard ratios was described in detail in Methods section under "Sensitivity Signature Extraction". In this section, as well as in the caption of Figure 1, it is explicitly stated that the hazard ratios used in this context quantify *in vitro* survival differences rather than patient survival.

3. *The graphs of IC50 distributions (presented like Kaplan-Meier plots) for each drug would be a valuable inclusion in the supplemental materials. Such a graph was shown in Scarborough 2023 in Figure 3C for cisplatin; the same should be shown here for each of the other 9 chemotherapies.*

   These plots were included in Supplemental Figure 3.

4. *One last point would be a valuable addition to the discussion, regarding the comparison of many drugs, which was not relevant to this group's 2023 article on cisplatin only. Given some set of drugs to choose between for some cancer type, in general these drugs will not have equal overall clinical efficacy; some drugs will be more effective than others on average. Consider for example 2 drugs, where one has a 50% response rate, and the other has a 20% response rate. If a patient is predicted to have 'median' sensitivity to the first drug, but 'top 30%' sensitivity to the second drug, then rank order may predict the second drug to be better, but when considering differences among drugs in overall response rates, such a patient would be better off receiving the first drug with the higher response rate. This complication needn't be*

solved in this pre-clinical proof-of-concept study, but its relevance to future clinical applications of choosing between drugs deserves discussion.

This would certainly be important information to account for as our approach for personalizing chemotherapy develops! As pointed out in the Discussion section, the formation of some sort of sensitivity score threshold would be critical for identifying what drugs would truly be effective for a patient, and not just identifying what drugs work *relatively* better than others. Utilizing information about overall clinical efficacy would be very useful for creating sensitivity score thresholds, and we've added comments on this in the Discussion.
