# Supplementary material for: Personalizing chemotherapy drug selection using a novel transcriptomic chemogram
Source: PLoS Comput Biol. 2025 Sep 10;21(9):e1013417. doi: 10.1371/journal.pcbi.1013417 (PMC12449002; doi:10.1371/journal.pcbi.1013417)
Supplement: Attachment — Submitted filename: Review of revision.docx [file pcbi.1013417.s013.docx]

The revised manuscript fully addresses the comments of my initial review, which were chiefly about some further explanations of methods and disease settings in which the current data suggest the greatest potential for applicability. The revised article accomplishes these tasks very nicely. The revised discussion especially makes a compelling case for the clinical need and the potential impact of chemograms and related approaches for precision oncology, which stand out as a very distinct approach, of use for different drugs, when compared with genome sequence-based inhibition of oncogenes. It is true, as hinted in comments by reviewer 1 also, that improved predictive accuracy remains desirable, but the breadth of application of this study and its inclusion of complete source data and software for reproducibility makes this paper a valuable contribution to progress on the important and tough challenge of precision cancer medicine.
